# Chlorido{1-[(dimethylamino)methyl]ferrocenyl-κ^2^ *C* ^2^,*N*}(tri­phenyl­stibine-κ*Sb*)palladium(II)

**DOI:** 10.1107/S1600536813015109

**Published:** 2013-06-08

**Authors:** Diego Pérez, Pankaj Sharma, Manju Sharma, Simón Hernández

**Affiliations:** a Instituto de Química, Universidad Nacional Autónoma de México, Circuito Exterior s/n Ciudad Universitaria, 04510, México D.F., México; b Ingeniería Bioquímica, Instituto Tecnológico Superior de Atlixco, Atlixco, Puebla, México

## Abstract

In the title compound, [FePdCl(C_5_H_5_)(C_8_H_11_N)(C_18_H_15_Sb)], obtained by reaction of diphen­yl(*N*,*N*-di­methyl­amino­methyl­ferrocen­yl)stibine with sodium tetra­chlorido­palladate(II) in acetone, the Pd^II^ atom is coordinated in a slightly distorted square-planar geometry by a C atom of the ferrocenyl ring, and by N, Cl and Sb atoms. The Sb and N atoms are *trans* to each other.

## Related literature

For the use of 1,2-disubstituted ferrocenylphosphines as catalytic precursors, see: Sokolov *et al.* (2005 ▶); Zirakzadeh *et al.* (2012 ▶). For Pd—Sb bond lengths in related compounds, see: Mentes & Fawcett (2005 ▶).
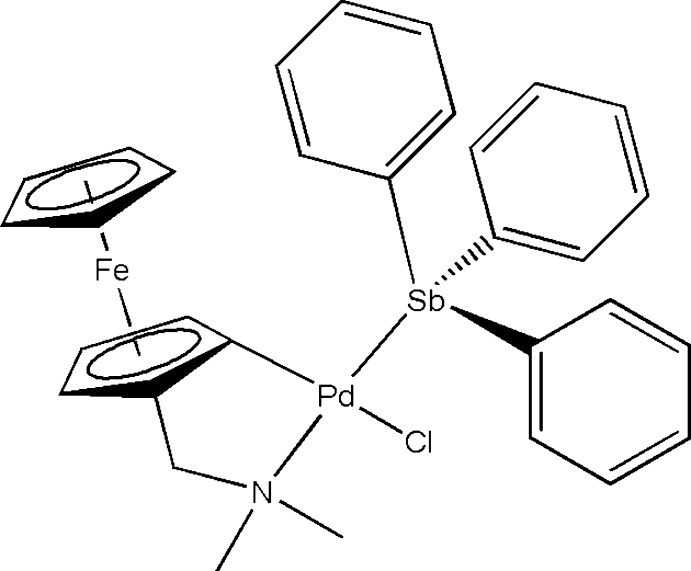


## Experimental

### 

#### Crystal data

[FePdCl(C_5_H_5_)(C_8_H_11_N)(C_18_H_15_Sb)]
*M* *_r_* = 737.02
Monoclinic, 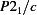

*a* = 10.3138 (6) Å
*b* = 19.8865 (12) Å
*c* = 13.7584 (9) Å
β = 92.984 (1)°
*V* = 2818.1 (3) Å^3^
*Z* = 4
Mo *K*α radiation
μ = 2.21 mm^−1^
*T* = 291 K
0.36 × 0.12 × 0.10 mm

#### Data collection

Bruker SMART APEX CCD area-detector diffractometer
Absorption correction: analytical (*SADABS*; Bruker, 2007 ▶) *T*_min_ = 0.490, *T*_max_ = 0.797
23494 measured reflections
5158 independent reflections
4107 reflections with *I* > 2σ(*I*)
*R*_int_ = 0.048

#### Refinement

*R*[*F* ^2^ > 2σ(*F* ^2^)] = 0.032
*wR*(*F* ^2^) = 0.063
*S* = 0.93
4963 reflections
327 parameters
H-atom parameters constrained
Δρ_max_ = 0.77 e Å^−3^
Δρ_min_ = −0.31 e Å^−3^

### 

Data collection: *SMART* (Bruker, 2007 ▶); cell refinement: *SAINT* (Bruker, 2007 ▶); data reduction: *SAINT*; program(s) used to solve structure: *SHELXTL* (Sheldrick, 2008 ▶); program(s) used to refine structure: *SHELXTL*; molecular graphics: *SHELXTL*; software used to prepare material for publication: *SHELXTL*.

## Supplementary Material

Crystal structure: contains datablock(s) I, global. DOI: 10.1107/S1600536813015109/vn2072sup1.cif

Structure factors: contains datablock(s) I. DOI: 10.1107/S1600536813015109/vn2072Isup2.hkl

Additional supplementary materials:  crystallographic information; 3D view; checkCIF report

## Figures and Tables

**Table 1:** Selected bond lengths (Å)

Sb1—C14	2.127 (4)
Sb1—C20	2.120 (4)
Sb1—C26	2.119 (4)
Sb1—Pd1	2.4853 (4)
Pd1—C1	1.973 (4)
Pd1—Cl1	2.3952 (10)
Pd1—N1	2.174 (3)

**Fig. 1.**
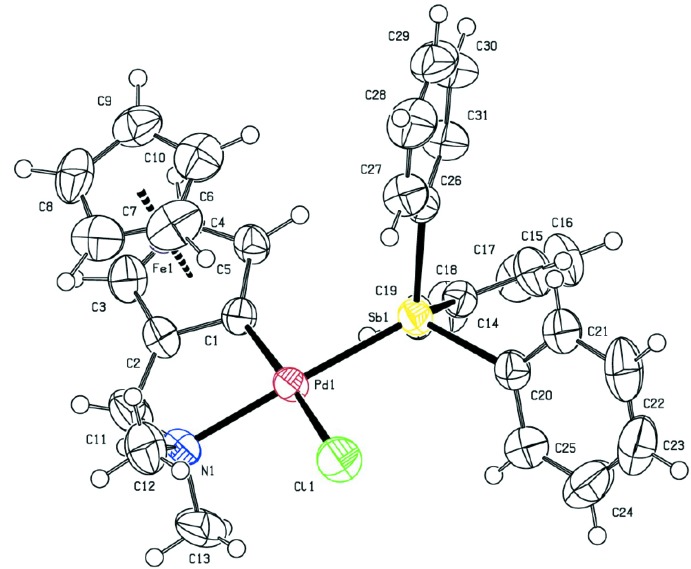
The title molecule with numbering scheme. The displacement ellipsoids are shown at the 50% probability level.

## supplementary crystallographic information

### Comment

1,2-disubstituted ferrocenylphosphines are used as catalytic precursors (Sokolov *et al.*, 2005; Zirakzadeh *et al.*, 2012). In an attempt to synthesize *cis*-dichloro[diphenyl(2-*N*,*N*-dimethylaminomethyl ferrocenyl)-stibino-N,Sb)]palladium(II), [PdCl(C_10_H_8_Fe)(C_3_H_8_N)(C_18_H_15_Sb)] was obtained. This paper describes the crystal structure of a new compound *viz*. chloro-(2-*N*,*N*-dimethylaminomethylferrocenyl)-(triphenylstibino) palladium (II), (Fig. 1), which was grown from CHCl_3_ and hexane at room temperature. In the crystal structure, palladium is in a slightly distorted square-planar environment bound to a chlorine, the antimony, the nitrogen and the C(1) atom of the ferocenyl unit. The compound contains a bicyclic system, which is formed by the substituted pentagonal ring of the ferrocenyl fragment and a five-membered palladacycle which has an envelope-like conformation. The Pd—Sb bond length in this compound is 2.4853 (4) Å which compares well with the literature values for similar complexes of palladium (Mentes & Fawcett, 2005). The *cis* angles at Pd are close to the expected value of 90°, with the most noticeable distortion being the N—Pd—Cl angle of 93.71 (7)°, as a result of chelation (Fig. 1). The distance between the Fe and the Pd is 3.4836 (7)° for this compound, thus suggesting that there is interaction between the two metals. The two pentagonal rings of the ferrocenyl moiety are nearly parallel (tilt angle 1.74°).

### Experimental

The title compound was obtained as follows: a solution of sodium tetrachloropalladate (0.294 g, 1 mmol) in acetone-water mixture (5 ml, 50–50%) is added to a solution of diphenyl(*N*,*N*-dimethyl aminomethylferrocenyl)stibine (0.519 g, 1 mmol) in acetone(10 ml). The mixture was stirred overnight. A red powder was obtained, which after recrystallization from a chloroform-hexane solvent mixture afforded chloro-(2-*N*,*N*-dimethylaminomethylferrocenyl)(triphenylstibino) palladium (II) as red crystals in 40% yield.

### Refinement

The H-atoms were placed geometrically (C—H = 0.93–0.98 Å), and refined using a riding model with *U*_iso_(H) = 1.2U_eq_ of the carrier atom.

### Crystal data

**Table:**

[FePdCl(C_5_H_5_)(C_8_H_11_N)(C_18_H_15_Sb)]	*F*(000) = 1456
*M**_r_* = 737.02	*D*_x_ = 1.737 Mg m^−^^3^
Monoclinic, *P*2_1_/*c*	Mo *K*α radiation, λ = 0.71073 Å
*a* = 10.3138 (6) Å	Cell parameters from 5485 reflections
*b* = 19.8865 (12) Å	θ = 2.2–30.8°
*c* = 13.7584 (9) Å	µ = 2.21 mm^−^^1^
β = 92.984 (1)°	*T* = 291 K
*V* = 2818.1 (3) Å^3^	Prism, red
*Z* = 4	0.36 × 0.12 × 0.10 mm

### Data collection

**Table:**

Bruker SMART APEX CCD area-detector diffractometer	5158 independent reflections
Radiation source: fine-focus sealed tube	4107 reflections with *I* > 2σ(*I*)
Graphite monochromator	*R*_int_ = 0.048
Detector resolution: 0.83 pixels mm^-1^	θ_max_ = 25.4°, θ_min_ = 1.8°
ω scans	*h* = −12→12
Absorption correction: analytical (*SADABS*; Bruker, 2007)	*k* = −23→23
*T*_min_ = 0.490, *T*_max_ = 0.797	*l* = −16→16
23494 measured reflections	

### Refinement

**Table:**

Refinement on *F*^2^	0 restraints
Least-squares matrix: full	Hydrogen site location: inferred from neighbouring sites
*R*[*F*^2^ > 2σ(*F*^2^)] = 0.032	H-atom parameters constrained
*wR*(*F*^2^) = 0.063	*w* = 1/[σ^2^(*F*_o_^2^) + (0.027*P*)^2^] where *P* = (*F*_o_^2^ + 2*F*_c_^2^)/3
*S* = 0.93	(Δ/σ)_max_ = 0.002
4963 reflections	Δρ_max_ = 0.77 e Å^−^^3^
327 parameters	Δρ_min_ = −0.31 e Å^−^^3^

### Special details

**Table:**

Geometry. All e.s.d.'s (except the e.s.d. in the dihedral angle between two l.s. planes) are estimated using the full covariance matrix. The cell e.s.d.'s are taken into account individually in the estimation of e.s.d.'s in distances, angles and torsion angles; correlations between e.s.d.'s in cell parameters are only used when they are defined by crystal symmetry. An approximate (isotropic) treatment of cell e.s.d.'s is used for estimating e.s.d.'s involving l.s. planes.
Refinement. Refinement of *F*^2^ against ALL reflections. The weighted *R*-factor *wR* and goodness of fit *S* are based on *F*^2^, conventional *R*-factors *R* are based on *F*, with *F* set to zero for negative *F*^2^. The threshold expression of *F*^2^ > σ(*F*^2^) is used only for calculating *R*-factors(gt) *etc*. and is not relevant to the choice of reflections for refinement. *R*-factors based on *F*^2^ are statistically about twice as large as those based on *F*, and *R*- factors based on ALL data will be even larger.

### Fractional atomic coordinates and isotropic or equivalent isotropic displacement parameters (Å^2^)

**Table:**

	*x*	*y*	*z*	*U*_iso_*/*U*_eq_	
Sb1	0.76605 (2)	0.06765 (2)	0.80847 (2)	0.03930 (8)	
Pd1	0.63076 (3)	0.12392 (2)	0.67766 (2)	0.03893 (9)	
Fe1	0.83892 (6)	0.11299 (3)	0.48685 (4)	0.04535 (15)	
Cl1	0.55085 (11)	0.20492 (5)	0.78865 (8)	0.0617 (3)	
N1	0.5010 (3)	0.15681 (15)	0.5573 (2)	0.0436 (8)	
C1	0.7077 (4)	0.06914 (18)	0.5758 (3)	0.0404 (9)	
C2	0.6501 (4)	0.08030 (19)	0.4804 (3)	0.0468 (10)	
C3	0.7239 (4)	0.0457 (2)	0.4126 (3)	0.0539 (11)	
H3	0.7062	0.0447	0.3419	0.065*	
C4	0.8258 (4)	0.01262 (19)	0.4639 (3)	0.0525 (11)	
H4	0.8918	−0.0156	0.4353	0.063*	
C5	0.8176 (4)	0.02703 (18)	0.5641 (3)	0.0442 (10)	
H5	0.8773	0.0107	0.6166	0.053*	
C6	0.8900 (5)	0.2057 (2)	0.5419 (4)	0.0734 (14)	
H6	0.8525	0.2275	0.5978	0.088*	
C7	0.8440 (5)	0.2115 (2)	0.4449 (4)	0.0740 (15)	
H7	0.7699	0.2386	0.4204	0.089*	
C8	0.9262 (5)	0.1721 (2)	0.3882 (3)	0.0718 (14)	
H8	0.9191	0.1669	0.3173	0.086*	
C9	1.0205 (4)	0.1429 (2)	0.4515 (4)	0.0644 (13)	
H9	1.0911	0.1133	0.4329	0.077*	
C10	0.9961 (5)	0.1630 (2)	0.5460 (4)	0.0690 (13)	
H10	1.0469	0.1499	0.6052	0.083*	
C11	0.5280 (4)	0.1194 (2)	0.4671 (3)	0.0677 (13)	
H11A	0.4564	0.0892	0.4506	0.081*	
H11B	0.5352	0.1509	0.4138	0.081*	
C12	0.5130 (5)	0.2297 (2)	0.5424 (3)	0.0702 (13)	
H12A	0.5993	0.2400	0.5240	0.105*	
H12B	0.4962	0.2528	0.6017	0.105*	
H12C	0.4514	0.2438	0.4918	0.105*	
C13	0.3667 (4)	0.1431 (3)	0.5849 (4)	0.0756 (14)	
H13A	0.3515	0.1652	0.6452	0.113*	
H13B	0.3550	0.0955	0.5924	0.113*	
H13C	0.3064	0.1596	0.5350	0.113*	
C14	0.7714 (4)	−0.03917 (18)	0.8157 (3)	0.0402 (9)	
C15	0.8309 (4)	−0.0706 (2)	0.8955 (3)	0.0578 (12)	
H15	0.8687	−0.0448	0.9457	0.069*	
C16	0.8350 (5)	−0.1400 (2)	0.9019 (3)	0.0670 (13)	
H16	0.8766	−0.1608	0.9554	0.080*	
C17	0.7766 (4)	−0.1780 (2)	0.8279 (3)	0.0637 (13)	
H17	0.7804	−0.2247	0.8311	0.076*	
C18	0.7138 (4)	−0.1479 (2)	0.7506 (3)	0.0611 (12)	
H18	0.6717	−0.1739	0.7024	0.073*	
C19	0.7122 (4)	−0.07832 (19)	0.7435 (3)	0.0498 (10)	
H19	0.6709	−0.0579	0.6895	0.060*	
C20	0.7170 (4)	0.08795 (19)	0.9533 (3)	0.0446 (10)	
C21	0.8071 (4)	0.1086 (2)	1.0237 (3)	0.0597 (12)	
H21	0.8938	0.1127	1.0092	0.072*	
C22	0.7691 (7)	0.1234 (2)	1.1168 (3)	0.0816 (16)	
H22	0.8306	0.1384	1.1637	0.098*	
C23	0.6431 (7)	0.1163 (3)	1.1405 (4)	0.0837 (18)	
H23	0.6187	0.1259	1.2031	0.100*	
C24	0.5537 (6)	0.0950 (3)	1.0711 (4)	0.0837 (16)	
H24	0.4677	0.0896	1.0868	0.100*	
C25	0.5888 (4)	0.0814 (2)	0.9785 (3)	0.0634 (12)	
H25	0.5260	0.0675	0.9318	0.076*	
C26	0.9668 (4)	0.09096 (19)	0.8159 (3)	0.0405 (9)	
C27	1.0016 (4)	0.1584 (2)	0.8228 (3)	0.0579 (11)	
H27	0.9374	0.1913	0.8220	0.069*	
C28	1.1312 (5)	0.1770 (2)	0.8310 (3)	0.0716 (14)	
H28	1.1536	0.2222	0.8363	0.086*	
C29	1.2254 (5)	0.1295 (3)	0.8311 (3)	0.0741 (14)	
H29	1.3123	0.1422	0.8366	0.089*	
C30	1.1933 (4)	0.0630 (3)	0.8233 (4)	0.0746 (14)	
H30	1.2584	0.0306	0.8236	0.090*	
C31	1.0645 (4)	0.0436 (2)	0.8149 (3)	0.0592 (12)	
H31	1.0435	−0.0017	0.8086	0.071*	

### Atomic displacement parameters (Å^2^)

**Table:**

	*U*^11^	*U*^22^	*U*^33^	*U*^12^	*U*^13^	*U*^23^
Sb1	0.03905 (15)	0.04112 (15)	0.03734 (15)	0.00225 (12)	−0.00182 (11)	−0.00163 (12)
Pd1	0.03648 (17)	0.04065 (17)	0.03923 (17)	0.00368 (14)	−0.00218 (13)	−0.00211 (14)
Fe1	0.0529 (4)	0.0395 (3)	0.0439 (3)	0.0023 (3)	0.0053 (3)	0.0017 (3)
Cl1	0.0681 (7)	0.0604 (7)	0.0565 (7)	0.0136 (6)	0.0026 (6)	−0.0131 (5)
N1	0.0372 (18)	0.0446 (19)	0.048 (2)	0.0038 (15)	−0.0076 (15)	0.0045 (16)
C1	0.044 (2)	0.040 (2)	0.038 (2)	−0.0006 (18)	0.0023 (18)	−0.0011 (18)
C2	0.052 (3)	0.042 (2)	0.045 (2)	0.002 (2)	−0.008 (2)	−0.0012 (19)
C3	0.070 (3)	0.050 (3)	0.042 (2)	−0.003 (2)	−0.004 (2)	−0.011 (2)
C4	0.063 (3)	0.036 (2)	0.059 (3)	0.007 (2)	0.013 (2)	−0.001 (2)
C5	0.051 (2)	0.037 (2)	0.045 (2)	0.0072 (19)	0.0011 (19)	0.0036 (18)
C6	0.084 (4)	0.047 (3)	0.092 (4)	−0.013 (3)	0.026 (3)	−0.011 (3)
C7	0.067 (3)	0.044 (3)	0.112 (5)	0.000 (2)	0.014 (3)	0.027 (3)
C8	0.094 (4)	0.067 (3)	0.055 (3)	−0.019 (3)	0.010 (3)	0.019 (3)
C9	0.059 (3)	0.056 (3)	0.080 (4)	−0.004 (2)	0.021 (3)	0.005 (3)
C10	0.070 (3)	0.063 (3)	0.075 (4)	−0.018 (3)	0.004 (3)	−0.004 (3)
C11	0.063 (3)	0.084 (3)	0.054 (3)	0.018 (3)	−0.017 (2)	−0.014 (3)
C12	0.084 (4)	0.059 (3)	0.067 (3)	0.003 (3)	−0.006 (3)	0.016 (2)
C13	0.040 (3)	0.098 (4)	0.088 (4)	−0.007 (3)	−0.011 (2)	0.006 (3)
C14	0.042 (2)	0.041 (2)	0.038 (2)	−0.0042 (19)	0.0008 (18)	0.0003 (18)
C15	0.069 (3)	0.050 (3)	0.053 (3)	−0.006 (2)	−0.017 (2)	0.000 (2)
C16	0.079 (3)	0.055 (3)	0.064 (3)	−0.003 (3)	−0.019 (3)	0.017 (2)
C17	0.078 (3)	0.041 (2)	0.073 (3)	−0.003 (2)	0.011 (3)	0.006 (2)
C18	0.080 (3)	0.047 (3)	0.055 (3)	−0.008 (2)	−0.007 (2)	−0.005 (2)
C19	0.059 (3)	0.050 (3)	0.040 (2)	−0.007 (2)	−0.005 (2)	0.004 (2)
C20	0.052 (3)	0.040 (2)	0.042 (2)	0.007 (2)	0.001 (2)	−0.0006 (18)
C21	0.062 (3)	0.069 (3)	0.048 (3)	0.000 (2)	−0.002 (2)	0.001 (2)
C22	0.129 (5)	0.073 (4)	0.041 (3)	0.006 (4)	−0.015 (3)	−0.006 (3)
C23	0.131 (6)	0.072 (4)	0.050 (3)	0.031 (4)	0.024 (4)	0.011 (3)
C24	0.090 (4)	0.082 (4)	0.083 (4)	0.017 (3)	0.044 (4)	0.013 (3)
C25	0.057 (3)	0.073 (3)	0.061 (3)	0.002 (2)	0.006 (2)	0.001 (2)
C26	0.042 (2)	0.046 (2)	0.033 (2)	−0.005 (2)	0.0040 (17)	0.0003 (18)
C27	0.059 (3)	0.050 (3)	0.065 (3)	−0.002 (2)	0.008 (2)	0.000 (2)
C28	0.075 (4)	0.060 (3)	0.080 (4)	−0.029 (3)	0.006 (3)	0.002 (3)
C29	0.052 (3)	0.106 (4)	0.065 (3)	−0.021 (3)	0.008 (2)	−0.001 (3)
C30	0.044 (3)	0.086 (4)	0.094 (4)	0.010 (3)	0.005 (3)	−0.006 (3)
C31	0.050 (3)	0.053 (3)	0.074 (3)	0.000 (2)	0.002 (2)	−0.003 (2)

### Geometric parameters (Å, º)

**Table:**

Sb1—C14	2.127 (4)	C11—H11B	0.9700
Sb1—C20	2.120 (4)	C12—H12A	0.9600
Sb1—C26	2.119 (4)	C12—H12B	0.9600
Sb1—Pd1	2.4853 (4)	C12—H12C	0.9600
Pd1—C1	1.973 (4)	C13—H13A	0.9600
Pd1—Cl1	2.3952 (10)	C13—H13B	0.9600
Pd1—N1	2.174 (3)	C13—H13C	0.9600
Fe1—C4	2.024 (4)	C14—C15	1.378 (5)
Fe1—C3	2.029 (4)	C14—C19	1.380 (5)
Fe1—C5	2.031 (4)	C15—C16	1.383 (6)
Fe1—C10	2.035 (5)	C15—H15	0.9300
Fe1—C8	2.040 (4)	C16—C17	1.380 (6)
Fe1—C7	2.044 (4)	C16—H16	0.9300
Fe1—C9	2.048 (4)	C17—C18	1.356 (6)
Fe1—C2	2.051 (4)	C17—H17	0.9300
Fe1—C6	2.051 (4)	C18—C19	1.387 (5)
Fe1—C1	2.065 (4)	C18—H18	0.9300
N1—C12	1.470 (5)	C19—H19	0.9300
N1—C13	1.481 (5)	C20—C21	1.370 (5)
N1—C11	1.485 (5)	C20—C25	1.391 (6)
C1—C5	1.426 (5)	C21—C22	1.391 (6)
C1—C2	1.429 (5)	C21—H21	0.9300
C2—C3	1.413 (5)	C22—C23	1.363 (7)
C2—C11	1.484 (5)	C22—H22	0.9300
C3—C4	1.400 (5)	C23—C24	1.360 (7)
C3—H3	0.9800	C23—H23	0.9300
C4—C5	1.414 (5)	C24—C25	1.370 (6)
C4—H4	0.9800	C24—H24	0.9300
C5—H5	0.9800	C25—H25	0.9300
C6—C10	1.384 (6)	C26—C31	1.380 (5)
C6—C7	1.398 (7)	C26—C27	1.391 (5)
C6—H6	0.9800	C27—C28	1.385 (6)
C7—C8	1.418 (6)	C27—H27	0.9300
C7—H7	0.9800	C28—C29	1.356 (6)
C8—C9	1.398 (6)	C28—H28	0.9300
C8—H8	0.9800	C29—C30	1.366 (6)
C9—C10	1.395 (6)	C29—H29	0.9300
C9—H9	0.9800	C30—C31	1.382 (6)
C10—H10	0.9800	C30—H30	0.9300
C11—H11A	0.9700	C31—H31	0.9300
			
C26—Sb1—C20	101.15 (15)	Fe1—C6—H6	125.7
C26—Sb1—C14	101.14 (14)	C6—C7—C8	107.3 (5)
C20—Sb1—C14	98.81 (14)	C6—C7—Fe1	70.3 (3)
C26—Sb1—Pd1	116.53 (10)	C8—C7—Fe1	69.5 (2)
C20—Sb1—Pd1	116.27 (10)	C6—C7—H7	126.4
C14—Sb1—Pd1	119.72 (10)	C8—C7—H7	126.4
C1—Pd1—N1	83.09 (14)	Fe1—C7—H7	126.4
C1—Pd1—Cl1	171.20 (11)	C9—C8—C7	107.7 (4)
N1—Pd1—Cl1	93.71 (9)	C9—C8—Fe1	70.3 (3)
C1—Pd1—Sb1	91.96 (11)	C7—C8—Fe1	69.9 (3)
N1—Pd1—Sb1	170.67 (8)	C9—C8—H8	126.2
Cl1—Pd1—Sb1	92.29 (3)	C7—C8—H8	126.2
C4—Fe1—C3	40.42 (15)	Fe1—C8—H8	126.2
C4—Fe1—C5	40.83 (14)	C10—C9—C8	107.9 (4)
C3—Fe1—C5	68.41 (16)	C10—C9—Fe1	69.5 (3)
C4—Fe1—C10	126.1 (2)	C8—C9—Fe1	69.7 (3)
C3—Fe1—C10	163.0 (2)	C10—C9—H9	126.0
C5—Fe1—C10	107.91 (18)	C8—C9—H9	126.0
C4—Fe1—C8	119.50 (19)	Fe1—C9—H9	126.0
C3—Fe1—C8	108.15 (18)	C6—C10—C9	108.6 (5)
C5—Fe1—C8	153.73 (19)	C6—C10—Fe1	70.8 (3)
C10—Fe1—C8	67.3 (2)	C9—C10—Fe1	70.5 (3)
C4—Fe1—C7	154.6 (2)	C6—C10—H10	125.7
C3—Fe1—C7	120.8 (2)	C9—C10—H10	125.7
C5—Fe1—C7	163.7 (2)	Fe1—C10—H10	125.7
C10—Fe1—C7	67.2 (2)	C2—C11—N1	110.7 (3)
C8—Fe1—C7	40.63 (18)	C2—C11—H11A	109.5
C4—Fe1—C9	107.67 (17)	N1—C11—H11A	109.5
C3—Fe1—C9	126.17 (18)	C2—C11—H11B	109.5
C5—Fe1—C9	119.59 (18)	N1—C11—H11B	109.5
C10—Fe1—C9	39.96 (17)	H11A—C11—H11B	108.1
C8—Fe1—C9	40.01 (18)	N1—C12—H12A	109.5
C7—Fe1—C9	67.51 (19)	N1—C12—H12B	109.5
C4—Fe1—C2	67.99 (16)	H12A—C12—H12B	109.5
C3—Fe1—C2	40.53 (15)	N1—C12—H12C	109.5
C5—Fe1—C2	68.11 (15)	H12A—C12—H12C	109.5
C10—Fe1—C2	155.11 (18)	H12B—C12—H12C	109.5
C8—Fe1—C2	127.22 (19)	N1—C13—H13A	109.5
C7—Fe1—C2	109.27 (18)	N1—C13—H13B	109.5
C9—Fe1—C2	163.79 (19)	H13A—C13—H13B	109.5
C4—Fe1—C6	163.2 (2)	N1—C13—H13C	109.5
C3—Fe1—C6	155.6 (2)	H13A—C13—H13C	109.5
C5—Fe1—C6	126.6 (2)	H13B—C13—H13C	109.5
C10—Fe1—C6	39.61 (18)	C15—C14—C19	118.7 (4)
C8—Fe1—C6	67.3 (2)	C15—C14—Sb1	119.9 (3)
C7—Fe1—C6	39.92 (19)	C19—C14—Sb1	121.4 (3)
C9—Fe1—C6	66.82 (19)	C14—C15—C16	120.9 (4)
C2—Fe1—C6	121.72 (19)	C14—C15—H15	119.5
C4—Fe1—C1	68.63 (15)	C16—C15—H15	119.5
C3—Fe1—C1	68.64 (16)	C17—C16—C15	119.2 (4)
C5—Fe1—C1	40.73 (14)	C17—C16—H16	120.4
C10—Fe1—C1	120.16 (17)	C15—C16—H16	120.4
C8—Fe1—C1	164.44 (19)	C18—C17—C16	120.6 (4)
C7—Fe1—C1	126.83 (18)	C18—C17—H17	119.7
C9—Fe1—C1	154.07 (18)	C16—C17—H17	119.7
C2—Fe1—C1	40.64 (14)	C17—C18—C19	119.9 (4)
C6—Fe1—C1	108.92 (17)	C17—C18—H18	120.0
C12—N1—C13	107.7 (3)	C19—C18—H18	120.0
C12—N1—C11	110.8 (3)	C14—C19—C18	120.6 (4)
C13—N1—C11	109.9 (3)	C14—C19—H19	119.7
C12—N1—Pd1	110.5 (2)	C18—C19—H19	119.7
C13—N1—Pd1	107.2 (2)	C21—C20—C25	118.0 (4)
C11—N1—Pd1	110.6 (2)	C21—C20—Sb1	122.4 (3)
C5—C1—C2	106.4 (3)	C25—C20—Sb1	119.5 (3)
C5—C1—Pd1	139.1 (3)	C20—C21—C22	120.2 (5)
C2—C1—Pd1	113.8 (3)	C20—C21—H21	119.9
C5—C1—Fe1	68.3 (2)	C22—C21—H21	119.9
C2—C1—Fe1	69.1 (2)	C23—C22—C21	121.0 (5)
Pd1—C1—Fe1	119.20 (17)	C23—C22—H22	119.5
C3—C2—C1	108.6 (3)	C21—C22—H22	119.5
C3—C2—C11	131.0 (4)	C24—C23—C22	119.0 (5)
C1—C2—C11	120.3 (4)	C24—C23—H23	120.5
C3—C2—Fe1	68.9 (2)	C22—C23—H23	120.5
C1—C2—Fe1	70.2 (2)	C23—C24—C25	120.8 (5)
C11—C2—Fe1	129.6 (3)	C23—C24—H24	119.6
C4—C3—C2	108.2 (4)	C25—C24—H24	119.6
C4—C3—Fe1	69.6 (2)	C24—C25—C20	120.9 (5)
C2—C3—Fe1	70.6 (2)	C24—C25—H25	119.5
C4—C3—H3	125.9	C20—C25—H25	119.5
C2—C3—H3	125.9	C31—C26—C27	118.2 (4)
Fe1—C3—H3	125.9	C31—C26—Sb1	124.2 (3)
C3—C4—C5	108.3 (4)	C27—C26—Sb1	117.5 (3)
C3—C4—Fe1	70.0 (2)	C28—C27—C26	120.5 (4)
C5—C4—Fe1	69.8 (2)	C28—C27—H27	119.8
C3—C4—H4	125.8	C26—C27—H27	119.8
C5—C4—H4	125.8	C29—C28—C27	120.2 (4)
Fe1—C4—H4	125.8	C29—C28—H28	119.9
C4—C5—C1	108.5 (3)	C27—C28—H28	119.9
C4—C5—Fe1	69.3 (2)	C28—C29—C30	120.3 (5)
C1—C5—Fe1	70.9 (2)	C28—C29—H29	119.9
C4—C5—H5	125.7	C30—C29—H29	119.9
C1—C5—H5	125.7	C29—C30—C31	120.3 (5)
Fe1—C5—H5	125.7	C29—C30—H30	119.9
C10—C6—C7	108.5 (5)	C31—C30—H30	119.9
C10—C6—Fe1	69.5 (3)	C26—C31—C30	120.6 (4)
C7—C6—Fe1	69.8 (3)	C26—C31—H31	119.7
C10—C6—H6	125.7	C30—C31—H31	119.7
C7—C6—H6	125.7		
			
C5—C1—C2—C3	−0.1 (4)	C7—C6—C10—Fe1	−59.0 (3)
Pd1—C1—C2—C3	172.1 (3)	C8—C9—C10—C6	−1.6 (5)
Fe1—C1—C2—C3	58.4 (3)	Fe1—C9—C10—C6	−60.9 (3)
C5—C1—C2—C11	176.5 (4)	C8—C9—C10—Fe1	59.3 (3)
Pd1—C1—C2—C11	−11.3 (5)	C3—C2—C11—N1	−169.5 (4)
Fe1—C1—C2—C11	−125.0 (4)	C1—C2—C11—N1	14.8 (6)
C5—C1—C2—Fe1	−58.4 (2)	Fe1—C2—C11—N1	−73.8 (5)
Pd1—C1—C2—Fe1	113.7 (2)	C12—N1—C11—C2	112.3 (4)
C1—C2—C3—C4	0.5 (5)	C13—N1—C11—C2	−128.8 (4)
C11—C2—C3—C4	−175.6 (4)	Pd1—N1—C11—C2	−10.6 (4)
Fe1—C2—C3—C4	59.7 (3)	C19—C14—C15—C16	2.1 (6)
C1—C2—C3—Fe1	−59.2 (3)	Sb1—C14—C15—C16	179.7 (3)
C11—C2—C3—Fe1	124.7 (5)	C14—C15—C16—C17	−1.1 (7)
C2—C3—C4—C5	−0.8 (5)	C15—C16—C17—C18	−1.2 (7)
Fe1—C3—C4—C5	59.5 (3)	C16—C17—C18—C19	2.6 (7)
C2—C3—C4—Fe1	−60.3 (3)	C15—C14—C19—C18	−0.7 (6)
C3—C4—C5—C1	0.8 (4)	Sb1—C14—C19—C18	−178.3 (3)
Fe1—C4—C5—C1	60.3 (3)	C17—C18—C19—C14	−1.6 (7)
C3—C4—C5—Fe1	−59.6 (3)	C25—C20—C21—C22	−1.1 (6)
C2—C1—C5—C4	−0.4 (4)	Sb1—C20—C21—C22	177.9 (3)
Pd1—C1—C5—C4	−169.4 (3)	C20—C21—C22—C23	1.5 (7)
Fe1—C1—C5—C4	−59.4 (3)	C21—C22—C23—C24	−0.6 (8)
C2—C1—C5—Fe1	58.9 (3)	C22—C23—C24—C25	−0.6 (8)
Pd1—C1—C5—Fe1	−110.1 (4)	C23—C24—C25—C20	1.0 (8)
C10—C6—C7—C8	−1.1 (5)	C21—C20—C25—C24	−0.2 (7)
Fe1—C6—C7—C8	−60.0 (3)	Sb1—C20—C25—C24	−179.1 (4)
C10—C6—C7—Fe1	58.9 (3)	C31—C26—C27—C28	−1.6 (6)
C6—C7—C8—C9	0.1 (5)	Sb1—C26—C27—C28	178.0 (3)
Fe1—C7—C8—C9	−60.4 (3)	C26—C27—C28—C29	0.8 (7)
C6—C7—C8—Fe1	60.5 (3)	C27—C28—C29—C30	0.0 (8)
C7—C8—C9—C10	0.9 (5)	C28—C29—C30—C31	0.1 (8)
Fe1—C8—C9—C10	−59.2 (3)	C27—C26—C31—C30	1.7 (6)
C7—C8—C9—Fe1	60.1 (3)	Sb1—C26—C31—C30	−177.8 (3)
C7—C6—C10—C9	1.7 (5)	C29—C30—C31—C26	−1.0 (7)
Fe1—C6—C10—C9	60.7 (3)		
